# Athlete's Heart in Asian Military Males: The CHIEF Heart Study

**DOI:** 10.3389/fcvm.2021.725852

**Published:** 2021-09-29

**Authors:** Pang-Yen Liu, Kun-Zhe Tsai, Joao A. C. Lima, Carl J. Lavie, Gen-Min Lin

**Affiliations:** ^1^Department of Internal Medicine, Hualien Armed Forces General Hospital, Hualien City, Taiwan; ^2^Department of Internal Medicine, Tri-Service General Hospital and National Defense Medical Center, Taipei, Taiwan; ^3^Department of Dentistry, Tri-Service General Hospital and National Defense Medical Center, Taipei, Taiwan; ^4^Departments of Cardiology and Radiology, Johns Hopkins University School of Medicine, Baltimore, MD, United States; ^5^Ochsner Clinical School, John Ochsner Heart and Vascular Institute, The University of Queensland School of Medicine, New Orleans, LA, United States

**Keywords:** Asian athletes, cardiac remodeling, endurance exercise performance, muscular strength exercise, left ventricular diastolic function

## Abstract

**Background:** Elite athlete's heart is characterized by a greater left ventricular mass indexed by body surface area (LVMI) and diastolic function; however previous studies are mainly conducted in non-Asian athletes compared to sedentary controls.

**Methods:** This study included 1,388 male adults, aged 18–34 years, enrolled in the same unified 6-month physical training program in Taiwan. During the midterm exams of 2020, all trainees completed a 3-km run (endurance) test, and 577 were randomly selected to attend a 2-min push-up (muscular strength) test. Elite athletes were defined as the performance of each exercise falling one standard deviation above the mean (16%). Cardiac structure and function were measured by echocardiography and compared between elite and non-elite athletes. Multiple logistic regression analysis was used to determine the independent predictors of elite athlete status at each exercise modality.

**Results:** As compared to non-elite controls, elite endurance athletes had greater LVMI (84.4 ± 13.6 vs. 80.5 ± 12.9 g/m^2^, *p* < 0.001) and lateral mitral E'/A' ratio (2.37 ± 0.73 vs. 2.22 ± 0.76, *p* < 0.01) with lower late diastolic A' (7.77 ± 2.16 vs. 8.30 ± 3.69 cm/s, *p* = 0.03). Elite strength athletes had greater LVMI (81.8 ± 11.4 vs. 77.5 ± 12.1, *p* = 0.004) and lateral mitral E'/A' ratio (2.36 ± 0.70 vs. 2.11 ± 0.71, *p* < 0.01) with a greater early diastolic E' (19.30 ± 4.06 vs. 18.18 ± 4.05 cm/s, *p* = 0.02). Greater LVMI and lower heart rate were independent predictors of elite endurance athletes [odds ratio (OR) and 95% confidence intervals: 1.03 (1.02, 1.04) and 0.96 (0.95, 0.98), respectively]. Greater LVMI, lateral mitral E'/A' ratio and right ventricular systolic pressure were independent predictors of elite strength athletes [OR: 1.03 (1.01, 1.05), 1.50 (1.06, 2.12), and 1.12 (1.05, 1.19), respectively].

**Conclusions:** Cardiac structural and functional characteristics differ between endurance and strength elite athletes. While greater LVMI predicts elite status in both groups of Asian athletes, consistent with findings from Western elite athletes, greater diastolic function, and right ventricular systolic pressure characterize strength elite athletes, while lower heart rate at rest predicts endurance elite athletic status.

## Introduction

Aerobic and anaerobic fitness have been associated with cardiovascular (CV) health and mortality in the general population (1, 2). The performance in endurance and muscular strength exercises correlated well with aerobic and anaerobic fitness can modulate cardiac structure remodeling and diastolic left ventricular (LV) function. Prior studies have shown that elite athletes, such as Olympic athletes and US football players, had greater LV mass index (LVMI) and diastolic function measured by transthoracic echocardiography (3–5). In summary, elite athletes who expertise endurance exercise, or muscular strength exercise, or both had a greater LVMI than sedentary individuals or the reference values according to age suggested by the U.S. and European echocardiographic societies (6). With regard to the LV diastolic function, the E/A ratio evaluated by mitral inflow Doppler is slightly enhanced or normal in athletes compared to sedentary controls (5–7). As the LV diastolic function is assessed by tissue Doppler imaging of septal or lateral wall motion, the E'/A' ratio is significantly greater in athletes for an enhanced peak early E' or a reduced late atrial A' tissue velocity than controls (6–8). Currently, these findings for athlete's heart are mainly from the Western Countries.

Previous reports have revealed racial differences in the physiologically cardiac adaptions to regular exercise and to LV pressure overload (9–11). For given levels of physical training, athletes of African/Afro-Caribbean descent display more marked cardiac structure changes than do Caucasian athletes (10, 11), possibly due in part to genetic variations. However, there have been rare studies investigating CV health in Asian athletes. Moreover, prior studies compared elite athletes to sedentary controls but rarely to those in a similar training program whether the marked cardiac adaptations in elite athletes are also observed in physically active individuals are unknown (12). Therefore the aim of this study is to investigate the cardiac structure and function of elite athletes from a military population of physically active males in Taiwan.

## Methods

### Study Population

The cardiorespiratory fitness and hospitalization events in armed forces study (CHIEF Heart Study) included 1,388 military males, aged 18–34 years, from the ROC Army Huadong Defense Command Base, in Taiwan in January 2020 (13–17). All participants underwent the annual health examination, and self-reported a questionnaire for their habits of toxic substance use including tobacco smoking and alcohol consumption (active vs. former and never) in the Hualien Armed Forces General Hospital of Eastern Taiwan. In the Base, all military males have to receive a unified physical training program for two 3-km runs at 6:00 a.m. and at 16:00 p.m., respectively, along with their Company members led by the Captain within 20 min daily. In addition, all military males perform at least 20 successive push-ups and 20 successive sit-ups in order after each run in unlimited time. In July, 2020, all participants attended the midterm exams for an evaluation of their physical fitness. Of these, 1,388 participants received a 3-km run for examining their endurance capacity, and 577 participants randomly selected from the overall subjects by the Commander for a 2-min push-up test 1 week later to examine their muscular strength capacity. After exams, all participants carried out a 12-lead electrocardiography (ECG) and transthoracic echocardiography (TTE) for assessing their cardiac structure and function before August 31, 2020.

### Anthropometric Measurements

Measurements of body height and body weight of each study participant were performed in a standing position. Body mass index was defined as body weight (kg) divided by body height squared (m^2^). Mean blood pressure (BP) of each participant at rest was defined as (2 × diastolic BP + 1 × systolic BP) divided by 3. Body surface area was calculated as 0.20247 × body height (m)^0.725^ × body weight (kg)^0.425^ according to the Dubois formula (18).

### Physical Fitness Measurements

The endurance capacity of each study participant was evaluated by time for a 3-km run. All examinees did not carry any heavy objects and the test was performed on a flat playground at the Military Physical Training and Testing Center in Hualien, Taiwan. The aerobic exercise test was held outdoor at 16:00 p.m. only when the product of outdoor temperature (°C) and relative humidity (%) × 0.1 was <40 and the weather was not raining.

The muscular strength capacity of each participant was investigated by the 2-min push-up performance (2, 19). The upward and downward movements of push-ups of each examinee were performed on a sponge pad, and were scored only if the examinee's back and buttock line got the baseline peak and bottom levels set by the infrared sensors of a computerized scoring system during the priming period. However, the push-up test was aborted when any parts of the examinee's body except the hands and foot touched the pad before the time ran out (2 min).

### TTE Measurements

The TTE using a 1–5 MHz transducer (iE33; Philips Medical Systems, Andover, MA, USA) was performed by the same experienced technician under the supervision by the certificated cardiologist at the Hualien-Armed Forces General Hospital. Measurements of cardiac structure such as LV wall thickness and chamber dimensions were based on the recommendations of the American Society of Echocardiography (20). LV mass was calculated at end diastole according to the corrected formula proposed by Fernandes et al. (21). LV mass = 0.8 × {1.04 × [(LV internal diameter (LVIDd) + posterior wall thickness + interventricular septal thickness]^3^ – LVIDd^3^} + 0.6. LV hypertrophy for males was defined as the LV mass indexed by body surface area (LVMI) ≥88 g/m^2^ based on the Dubois formula (16, 22). Right ventricular (RV) hypertrophy for males was defined as the anterior RV wall thickness in parasternal long-axis window >5.2 mm (17). LV diastolic function was assessed by mitral inflow power Doppler for the early diastolic E wave, the late diastolic A wave related to atrial contraction and the E/A ratio, and assessed by tissue Doppler imaging for the lateral mitral annulus velocity of early diastolic E', the late diastolic A' and the E'/A' ratio. RV systolic pressure (RVSP) was assessed by the continuous wave Doppler in the four-chamber window.

### Statistical Analysis

Elite athletes were defined as the score of each exercise falling one standard deviation above the mean (16%), and the controls were the other physically active males not getting to the level of elite athletes in each exercise (84%). Demographic, anthropometric, ECG and TTE characteristics of the elite athletes and the non-elite controls were expressed as mean ± standard deviation for continuous variables and numbers (%) for categorical variables, respectively. Continuous variables were compared by analysis of variance (ANOVA) and categorical variables were compared by chi-square or Fisher's exact test. Dimensions of cardiac chambers and wall thickness were compared utilizing analysis of covariance (ANCOVA) with adjustment for body surface area. Multiple logistic regressions were used to determine the odds ratio (OR) of the TTE characteristics with the elite athletes to non-elite controls. In model 1, age, smoking, LVMI, RVSP, and lateral mitral E'/A' ratio were adjusted. In model 2, BMI was additionally adjusted. In model 3, mean BP was additionally adjusted. In model 4, heart rate was further adjusted. A two-tailed value of *P* < 0.05 was considered significant. All analyses were performed using SAS version 9.4 (SAS Institute, Cary, NC, USA). This study was approved by the Institutional Review Board of the Mennonite Christian Hospital (No. 16-05-008) in Taiwan, and written informed consent was obtained from all participants.

## Results

### Clinical Features and Laboratory Findings

There were 233 males (16.8%) classified as elite endurance athletes who spent <780 s for a 3-km run and the other 1,155 physically active males (93.2%) were classified as non-elite controls (Table 1). In addition, there were 78 males (13.5%) classified as elite strength athletes who performed more than 54 push-ups within 2 min and the other 499 physically active males (86.5%) were classified as non-elite controls. Elite endurance athletes had lower levels of body weight related anthropometrics, such as BMI and waist circumference (WC), systolic BP, fasting plasma glucose and low-density lipoprotein, a higher level of high-density lipoprotein, and a relatively better 2-min push-ups score (49.9 ± 12.3 vs. 45.5 ± 10.0). Elite strength athletes had a lower WC and a higher high-density lipoprotein, and a relatively better 3-km run score (833.2 ± 82.2 vs. 869.8 ± 81.6 s).

**Table 1:** Clinical characteristics of elite endurance and strength athletes and non-elite controls.

	**Participants attending a 3-KM run test** ** (*****N*** **=** **1,388)**			**Participants attending a 2-min push-up test** ** (*****N*** **=** **577)**		
**Clinical characteristics**	**Elite endurance athletes (≤780 s)** ** (*N* = 233)**	**Controls (>780 s) (*N* = 1,155)**	***P*-value**	**Elite strength athletes (≥54 times) (*N* = 78)**	**Controls** ** (<54 times)** ** (*N* = 499)**	***P*-value**
Age (years)	25.04 ± 3.64	25.21 ± 3.68	0.52	25.04 ± 3.75	25.22 ± 3.73	0.69
(Range: min–max)	(19–34)	(18–34)		(19–33)	(19–34)	
Height (cm)	171.65 ± 5.61	172.21 ± 5.76	0.17	171.32 ± 5.53	172.18 ± 5.62	0.21
Weight (kg)	68.43 ± 9.23	73.58 ± 12.08	<0.001	70.00 ± 9.61	72.70 ± 11.52	0.05
Body mass index (kg/m^2^)	23.20 ± 2.75	24.77 ± 3.62	<0.001	23.83 ± 3.07	24.49 ± 3.50	0.12
Body surface area (m^2^)	1.80 ± 0.13	1.87 ± 0.16	<0.001	1.82 ± 0.13	1.85 ± 0.16	0.04
Waist circumference (cm)	78.51 ± 6.97	83.10 ± 9.41	<0.001	79.76 ± 7.73	82.30 ± 8.90	0.01
Systolic blood pressure (mmHg)	116.07 ± 11.18	118.46 ± 12.30	0.006	116.36 ± 11.80	117.26 ± 11.85	0.53
Diastolic blood pressure (mmHg)	67.94 ± 8.41	69.15 ± 9.40	0.07	66.25 ± 8.69	68.24 ± 8.73	0.06
**Blood test**						
Creatinine (mg/dL)	0.94 ± 0.10	0.94 ± 0.11	0.96	0.96 ± 0.12	0.94 ± 0.10	0.11
Total cholesterol (mg/dL)	167.14 ± 31.83	167.68 ± 32.10	0.81	171.49 ± 34.32	167.93 ± 33.12	0.38
HDL-C (mg/dL)	52.55 ± 10.69	48.40 ± 9.72	<0.001	52.63 ± 11.24	48.92 ± 9.19	0.001
LDL-C (mg/dL)	98.40 ± 26.46	103.20 ± 28.77	0.01	103.90 ± 29.10	104.00 ± 30.38	0.97
Triglycerides (mg/dL)	86.51 ± 71.72	100.63 ± 76.39	0.009	93.35 ± 71.81	97.60 ± 67.10	0.60
Fasting glucose (mg/dL)	91.36 ± 8.32	92.95 ± 10.02	0.02	93.42 ± 10.09	93.09 ± 8.84	0.76
Current tobacco smoking	87 [37.3]	522 [45.2]	0.02	37 [48.7]	215 [43.1]	0.35
**Exercise performance**						
3-KM running (seconds)	748.08 ± 31.71	880.12 ± 74.30	<0.001	833.15 ± 82.75	869.84 ± 81.58	<0.001
2-min push-ups (numbers)^*^	49.95 ± 12.28	45.54 ± 10.04	<0.001	61.14 ± 10.92	43.88 ± 8.33	<0.001

*Continuous variables are expressed as mean ± SD (standard deviation), and categorical variables as n [%]*.

*HDL-C, high-density lipoprotein cholesterol; KM, kilometer; LDL-C, low-density lipoprotein cholesterol*.

* *N = 577*.

### ECG Features

In Table 2, elite endurance athletes had significantly slower heart rate (sinus bradycardia), greater QRS duration and axis, and a higher prevalence of ECG-based LV hypertrophy (70.8 vs. 54.8%, *p* < 0.001) according to the Soklow-Lyon voltage criterion (23). Elite strength athletes merely had a higher prevalence of the corrected QT interval prolongation >480 ms on the basis of the Bazett's formula (2.6 vs. 0.4%, *p* = 0.03) (24).

**Table 2:** Electrocardiographic characteristics of elite endurance and strength athletes and non-elite controls.

	**Participants attending a 3-KM run test** ** (*****N*** **=** **1,388)**			**Participants attending a 2-min push-up test** ** (*****N*** **=** **577)**		
**ECG characteristics**	**Elite endurance athletes (≤780 s)** ** (*N* = 233)**	**Controls (>780 s) (*N* = 1,155)**	***P*-value**	**Elite strength athletes (≥54 times) (*N* = 78)**	**Controls** ** (<54 times)** ** (*N* = 499)**	***P*-value**
Heart rate (beats/min)	61.93 ± 10.02	66.68 ± 10.58	<0.001	65.37 ± 12.05	66.03 ± 10.62	0.61
P duration (ms)	106.36 ± 11.78	105.90 ± 14.05	0.64	108.35 ± 11.75	105.56 ± 14.57	0.10
PR interval (ms)	156.77 ± 16.56	155.62 ± 18.81	0.38	156.41 ± 16.78	154.71 ± 19.93	0.47
QRS duration (ms)	99.95 ± 11.23	97.90 ± 10.95	0.03	98.62 ± 10.21	97.25 ± 10.02	0.26
QTc interval (ms)	387.02 ± 21.95	388.67 ± 22.81	0.30	392.23 ± 26.56	387.75 ± 21.73	0.10
QRS axis (degree)	71.06 ± 22.00	62.89 ± 31.61	<0.001	64.69 ± 25.05	65.00 ± 28.22	0.92
ECG based LVH (%)	165 [70.8]	633 [54.8]	<0.001	51 [65.4]	293 [58.7]	0.26
ECG based RVH (%)	35 [15.1]	177 [15.3]	0.92	13 [16.7]	81 [16.2]	0.92
Sinus bradycardia (%)	102 [43.8]	295 [25.5]	<0.001	28 [35.9]	137 [27.5]	0.12
Ectopic P rhythm (%)	19 [8.2]	108 [9.4]	0.56	5 [6.4]	55 [11.0]	0.21
Left atrial enlargement (%)	36 [15.5]	199 [17.2]	0.50	11 [14.1]	81 [16.2]	0.63
First degree atrioventricular block (%)	3 [1.3]	25 [2.2]	0.38	2 [2.6]	9 [1.8]	0.64
Left axis deviation (%)	0 [0.0]	18 [1.6]	0.05	2 [2.6]	3 [0.6]	0.08
Right axis deviation (%)	30 [12.9]	123 [10.6]	0.32	5 [6.4]	50 [10.0]	0.31
Complete right bundle branch block (%)	12 [5.1]	42 [3.6]	0.27	2 [2.6]	14 [2.8]	0.90
Incomplete right bundle branch block (%)	23 [9.9]	79 [6.8]	0.10	8 [10.3]	30 [6.0]	0.16
QTc prolongation > 480 ms (%)	1 [0.4]	7 [0.6]	0.74	2 [2.6]	2 [0.4]	0.03
T wave inversion (%)	6 [2.6]	37 [3.2]	0.61	2 [2.6]	15 [3.0]	0.83

*Categorical variables are expressed as N [%]*.

*ECG, electrocardiography; LVH, left ventricular hypertrophy; KM, kilometer; RVH, right ventricular hypertrophy*.

### TTE Findings

In Table 3, elite athletes and non-elite controls had similar chamber dimensions of left atrium, LV and RV in diastole, and similar LVM and RV wall thickness, except that elite endurance athletes had greater LV diastolic dimension with adjustment for body surface area. As compared to non-elite controls, elite endurance athletes had greater LVMI (84.4 ± 13.6 vs. 80.5 ± 12.9 g/m^2^, *p* < 0.001) and lateral mitral annulus E'/A' ratio (2.37 ± 0.73 vs. 2.22 ± 0.76, *p* < 0.01) with lower late diastolic A' (7.77 ± 2.16 vs. 8.30 ± 3.69 cm/s, *p* = 0.03). In contrast, elite strength athletes had greater LVMI (81.8 ± 11.4 vs. 77.5 ± 12.1 g/m^2^, *p* = 0.004), lateral mitral E'/A' ratio (2.36 ± 0.70 vs. 2.11 ± 0.71, *p* < 0.01) with greater early diastolic E' (19.30 ± 4.06 vs. 18.18 ± 4.05 cm/s, *p* = 0.02) and RVSP (29.60 ± 4.35 vs. 27.83 ± 3.71 mmHg, *p* < 0.001).

**Table 3:** Echocardiographic characteristics of elite endurance and strength athletes and non-elite controls.

	**Participants attending a 3-KM run test** ** (*****N*** **=** **1,388)**			**Participants attending a 2-min push-up test** ** (*****N*** **=** **577)**		
**Echocardiographic characteristics**	**Elite endurance athletes (≤780 s)** ** (*N* = 233)**	**Controls (>780 s) (*N* = 1,155)**	***P*-value**	**Elite strength athletes (≥54 times) (*N* = 78)**	**Controls** ** (<54 times)** ** (*N* = 499)**	***P*-value**
Aortic valve open (mm), PLAX	19.93 ± 1.98	20.03 ± 1.91	0.57^*^	20.53 ± 1.65	20.35 ± 1.86	0.61^*^
Aortic root dimension (mm), PALX	29.49 ± 3.23	29.68 ± 3.25	0.66^*^	30.13 ± 2.48	29.82 ± 2.74	0.19^*^
LV posterior wall (mm), PLAX^*^	8.44 ± 0.89	8.54 ± 0.94	0.31^*^	8.40 ± 0.76	8.29 ± 0.84	0.84^*^
LV internal dimension in diastole (mm), PLAX	50.03 ± 3.35	49.24 ± 3.42	0.003^*^	49.74 ± 3.19	49.07 ± 3.50	0.36^*^
Interventricular septum, (mm), PLAX	8.61 ± 0.95	8.74 ± 1.02	0.44^*^	8.53 ± 0.80	8.49 ± 0.89	0.45^*^
RV wall thickness (mm), PLAX^*^	4.72 ± 0.55	4.66 ± 0.62	0.98^*^	4.60 ± 0.67	4.70 ± 0.58	0.49^*^
RV outflow tract dimension in diastole (mm), PLAX	25.94 ± 3.84	27.19 ± 12.49	0.82^*^	26.47 ± 3.82	26.33 ± 3.96	0.45^*^
Left atrial dimension (mm), PLAX	32.55 ± 4.00	32.95 ± 3.93	0.21^*^	33.09 ± 3.40	32.83 ± 3.95	0.11^*^
LV mass (gm)	152.53 ± 28.43	151.05 ± 29.83	0.16	148.98 ± 23.54	144.45 ± 27.18	0.16
LV mass index (gm/m^2^)	84.44 ± 13.56	80.52 ± 12.94	<0.001	81.76 ± 11.36	77.51 ± 12.05	0.004
LV hypertrophy	11 [4.7]	24 [2.1]	0.01	1 [1.3]	5 [1.0]	0.82
RV hypertrophy	20 [8.6]	84 [7.3]	0.48	2 [2.6]	26 [5.2]	0.31
LV ejection fraction (%), PLAX	61.79 ± 5.03	61.97 ± 5.06	0.78	62.86 ± 5.36	62.43 ± 5.32	0.51
Tricuspid valve prolapse, PSAX	34 [20.4]	206 [26.6]	0.09	13 [41.9]	94 [51.1]	0.34
Aortic regurgitation ≥ mild grade	3 [1.3]	13 [1.1]	0.83	1 [1.3]	3 [0.6]	0.50
Mitral regurgitation ≥ mild grade	190 [81.5]	890 [77.1]	0.13	69 [88.5]	429 [86.0]	0.55
Pulmonary regurgitation ≥ mild grade	162 [69.5]	763 [66.1]	0.30	61 [78.2]	354 [70.9]	0.18
Tricuspid regurgitation ≥ mild grade	196 [84.1]	928 [80.3]	0.18	73 [93.6]	462 [92.6]	0.75
RV systolic pressure (mmHg)	27.68 ± 4.35	27.19 ± 4.36	0.27	29.60 ± 4.35	27.83 ± 3.71	<0.001
Mitral inflow power Doppler E-wave (m/s)	87.00 ± 14.13	86.21 ± 14.87	0.45	90.07 ± 13.63	87.41 ± 14.59	0.13
Mitral inflow power Doppler A-wave (m/s)	46.44 ± 9.59	48.69 ± 9.81	0.001	48.81 ± 9.42	49.31 ± 9.88	0.67
E/A ratio	1.94 ± 0.46	1.83 ± 0.46	0.001	1.91 ± 0.48	1.84 ± 0.47	0.20
Mitral lateral annulus tissue Doppler E' (cm/s)	17.33 ± 3.83	17.04 ± 3.75	0.29	19.30 ± 4.06	18.18 ± 4.05	0.02
Mitral lateral annulus tissue Doppler A' (cm/s)	7.77 ± 2.16	8.30 ± 3.69	0.03	8.51 ± 1.82	9.31 ± 4.95	0.16
E'/A' ratio	2.37 ± 0.73	2.22 ± 0.76	0.007	2.36 ± 0.70	2.11 ± 0.71	0.004

*Continuous variables are expressed as mean ± SD (standard deviation), and categorical variables as N [%]*.

*LV, left ventricle; RV, right ventricl; PLAX, echocardiographic parasternal long axis view; PSAX, echocardiographic parasternal short axis view*.

* *Analysis of covariance (ANCOVA) with adjustment for body surface area*.

### Echocardiographic Predictors of Elite Athletes

Table 4 demonstrates the results of multiple logistic regression analysis for the predictors of elite endurance and strength athletes, respectively. In model 1, greater LVMI and lateral mitral E'/A' were independent predictors of elite endurance athletes. However, the association for the lateral mitral E'/A' ratio was null after additionally controlling for BMI. In model 4, Greater LVMI, and lower heart rate and BMI were independent predictors of elite endurance athletes [odds ratio (OR): 1.03 (95% confidence intervals (CI): 1.02, 1.04), 0.96 (95% CI: 0.95, 0.98) and 0.85 (95% CI: 0.81, 0.90), respectively]. In contrast, greater LVMI, lateral mitral E'/A' and RVSP were independent predictors of elite strength athletes [OR: 1.03 (95% CI: 1.01, 1.05), 1.50 (95% CI: 1.06, 2.12) and 1.12 (95% CI: 1.05, 1.19), respectively] in model 4. Both BMI and heart rate were not independent predictors of elite strength athletes.

**Table 4:** Multivariable logistic regression analysis for elite endurance and strength athletes.

	**Model 1**			**Model 2**			**Model 3**			**Model 4**		
**Characteristics**	**OR**	**95% CI**	***P*-value**	**OR**	**95% CI**	***P*-value**	**OR**	**95% CI**	***P*-value**	**OR**	**95% CI**	***P*-value**
**Elite endurance athletes (*****N*** **=** **1,388)**												
Age	0.998	0.959–1.039	0.93	1.025	0.983–1.068	0.24	1.028	0.986–1.071	0.19	1.021	0.979–1.065	0.32
Tobacco smoking	0.704	0.525–0.943	0.01	0.694	0.515–0.936	0.01	0.687	0.509–0.927	0.01	0.759	0.560–1.029	0.07
LV mass index	1.023	1.012–1.034	<0.001	1.031	1.020–1.043	<0.001	1.032	1.021–1.044	<0.001	1.027	1.015–1.039	<0.001
RV systolic pressure	1.027	0.994–1.061	0.11	1.018	0.985–1.053	0.28	1.018	0.984–1.053	0.30	1.019	0.985–1.054	0.28
E'/A' ratio	1.291	1.072–1.556	0.007	1.163	0.958–1.411	0.12	1.159	0.955–1.407	0.13	1.076	0.883–1.312	0.46
Body mass index	–	–	–	0.847	0.806–0.890	<0.001	0.854	0.812–0.898	<0.001	0.852	0.809–0.897	<0.001
Mean blood pressure	–	–	–	–	–	–	0.987	0.970–1.004	0.12	0.991	0.974–1.008	0.29
Heart rate	–	–	–	–	–	–	–	–	–	0.964	0.949–0.979	<0.001
**Elite strength athletes (*****N*** **=** **577)**												
Age	1.013	0.947–1.085	0.70	1.023	0.954–1.096	0.52	1.028	0.958–1.103	0.44	1.030	0.960–1.106	0.40
Tobacco smoking	1.171	0.714–1.918	0.53	1.191	0.726–1.954	0.49	1.189	0.724–1.954	0.49	1.170	0.710–1.928	0.53
LV mass index	1.027	1.007–1.048	0.008	1.029	1.008–1.050	0.006	1.030	1.009–1.051	0.005	1.031	1.010–1.053	0.004
RV systolic pressure	1.124	1.056–1.197	<0.001	1.122	1.053–1.195	<0.001	1.121	1.052–1.195	<0.001	1.121	1.052–1.194	<0.001
E'/A' ratio	1.540	1.108–2.141	0.01	1.480	1.058–2.072	0.02	1.470	1.050–2.056	0.02	1.500	1.063–2.116	0.02
Body mass index	–	–	–	0.954	0.882–1.033	0.25	0.966	0.890–1.048	0.96	0.964	0.888–1.046	0.38
Mean blood pressure	–	–	–	–	–	–	0.981	0.952–1.012	0.22	0.980	0.951–1.011	0.20
Heart rate	–	–	–	–	–	–	–	–	–	1.007	0.983–1.032	0.57

*Data are presented as odds ratios (OR) and 95% CI (confidence intervals) using multiple logistic regression*.

*LV, left ventricle; RV, right ventricle*.

*Model 1 covariates included age, tobacco smoking and left ventricular mass index; Model 2 covariates included Model 1 covariates plus body mass index; Model 3 covariates included Model 2 covariates plus mean blood pressure; Model 4 covariates included Model 3 covariates plus heart rate*.

## Discussion

This study is the largest report to date to demonstrate the cardiac structure and function in Asian male athletes and compare them to physically active controls and to determine the predictors or eliteness based on endurance and muscular strength. The main findings in the present study were that elite endurance male athletes had greater LVMI and LV diastolic function which might be due to reduced heart rate. Greater LVMI, and lower heart rate and BMI were the independent predictors of falling in the elite endurance category. Elite strength male athletes also had greater LVMI and LV diastolic function; greater LVMI, lateral mitral E'/A' and RVSP were the independent predictors of being in the elite strength category.

Both elite endurance and strength athletes had greater LVMI, and endurance athletes had greater LV chamber size, which were consistent with the findings in prior studies (6, 25). Mechanisms for the LV hypertrophy induced by exercises have been proposed by a physiological compensation of cardiac muscle cells elongation in response to chronic hemodynamic overload that is regulated in part by the renin-angiotensin system (26, 27). The greater LV chamber size related to endurance exercises has been explained mainly by volume overload, but the LV chamber size is not enlarged in response to strength exercises (28). Although the LVMI of elite Asian male athletes in the present study was generally smaller than that reported in prior studies for Black and White male athletes, possibly due to the ethnical or racial differences in the genetic aspect, this study further confirmed the concept that LVMI could independently predict the performance of endurance and strength exercises in physically active males.

With regard to the enhanced LV diastolic function, the present study for elite Asian male athletes showed consistent results that elite endurance athletes had slightly greater E/A ratio due to a decrease in peak A wave velocity, and elite strength athletes had similar E/A ratio compared to their non-elite controls (11). In addition, with regard to the tissue Doppler image results, the greater lateral mitral E'/A' ratio in elite endurance athletes were due to a reduced A' velocity, whereas the greater lateral mitral E'/A' ratio in elite strength athletes were due to an increased peak E' velocity (6–8). In the multiple logistic regression model, the enhanced LV diastolic function in elite endurance athletes was due to a reduced resting heart rate, (29) and that in elite strength athletes might be related to an improved LV compliance. The reduced heart rate related to endurance exercise has been associated with an increase of parasympathetic tone (30). On the contrary, physically active Asian males regardless of endurance or strength exercise in the present study had a similar RVSP (25–29 mmHg) compared to non-Asian endurance athletes (26–27 mmHg), which was higher than that in sedentary controls in prior studies (16–22 mmHg) (25, 31). D'Andrea et al. reported that the RVSP was associated with RV chamber size and was higher in endurance athletes than strength athletes (20 mmHg) (25). However, the findings for right heart structure and function were contrary to the present study results whether these conflicts were because of racial/ethnic differences or for a presence of confounders such as the smoking habit which was not considered in prior studies requires further investigation.

### Study Strengths and Limitations

The major strength of the present study was that the male participants were enrolled in the same army camp in Taiwan where the training program was standardized. In addition, since the army base is a closed system, the living environment for the participants is very similar and their daily schedule, such as the wake up time, bed time, meal time and the frequency of sentry duty is unified. Third, the detailed information for the baseline confounders to both physical fitness and cardiac structure and function, such as tobacco smoking and alcohol consumption, were considered in this study, which could reduce a potential bias. By contrast, there were merely 36.8% of the overall males randomly selected to attend the push-up test, possibly leading to a selection bias, despite that the baseline characteristics between those with and without attending the push-up test were similar (Supplementary Table 1). Second, the endurance and muscular strength capacity for males were measured only by a short- to medium- distance run and a 2-min push-up test that the cardiac structure results might not be appropriately applied to other elite athletes for different kinds of exercise. Third, this study reported data from males only that might not be the same for females. Fourth, since the study was a cross-sectional design, the interval changes in cardiac structure and function could not be evaluated. At last, in many prior studies in Western Countries, athletes were defined to be at Olympic or Professional levels, and whether the present study results for the male athletes in military were consistent with more competitive male athletes in Taiwan needs further investigations.

## Conclusion

Our study uncovered that cardiac structural and functional characteristics differ between endurance and strength elite athletes in a physically active Asian male population. While greater LVMI predicts elite status in both groups of Asian athletes, consistent with findings from Western elite athletes, greater diastolic function and RV systolic pressure characterize strength elite athletes, while lower heart rate at rest predicts endurance elite athletic status.

## Data Availability Statement

The raw data supporting the conclusions of this article will be made available by the authors, without undue reservation.

## Ethics Statement

This study was approved by the Institutional Review Board of the Mennonite Christian Hospital (No. 16-05-008) in Taiwan, and written informed consent was obtained from all participants. The patients/participants provided their written informed consent to participate in this study.

## Author Contributions

P-YL and G-ML wrote the paper. K-ZT made the statistical analyses. JL and CL raised critical comments for the paper and edited the manuscript. G-ML was the principal investigator for the study. All authors contributed to the article and approved the submitted version.

## Funding

This study was supported by the Medical Affairs Bureau Ministry of National Defense and Hualien Armed Forces General Hospital, Taiwan, under the grants MND-MAB-110-148 and HAFGH-D-110008.

## Conflict of Interest

The authors declare that the research was conducted in the absence of any commercial or financial relationships that could be construed as a potential conflict of interest.

## Publisher's Note

All claims expressed in this article are solely those of the authors and do not necessarily represent those of their affiliated organizations, or those of the publisher, the editors and the reviewers. Any product that may be evaluated in this article, or claim that may be made by its manufacturer, is not guaranteed or endorsed by the publisher.
