# Supplementary material for: Athlete's Heart in Asian Military Males: The CHIEF Heart Study
Source: Front Cardiovasc Med. 2021 Sep 29;8:725852. doi: 10.3389/fcvm.2021.725852 (PMC8511640; doi:10.3389/fcvm.2021.725852)
Supplement: Supplementary file 1 [file Table_1.docx]

**Supplemental Table 1.** Clinical Characteristics of Participants With and Without Attending the Push-Up Test

|  | The Overall Participants  (N =1,388) | | |
| --- | --- | --- | --- |
| Clinical characteristics | Those for the push-up test  (N =577) | Those not for the push-up test  (N =811) | p-value |
| Age (years) | 25.19 ± 3.73 | 25.17 ± 3.63 | 0.91 |
| (Range: min – max) | 19 – 34 | 18 – 34 |  |
| Height (cm) | 172.06 ± 5.61 | 172.15 ± 5.82 | 0.78 |
| Weight (kg) | 72.33 ± 11.31 | 72.99 ± 12.14 | 0.30 |
| Body mass index (kg/m2) | 24.40 ± 3.45 | 24.58 ± 3.60 | 0.34 |
| Body surface area (m^2^) | 1.85 ± 0.15 | 1.86 ± 0.17 | 0.35 |
| Waist circumference (cm) | 81.95 ± 8.79 | 82.60 ± 9.49 | 0.19 |
| Systolic blood pressure (mmHg) | 117.14 ± 11.84 | 118.71 ± 12.33 | 0.01 |
| Diastolic blood pressure (mmHg) | 67.98 ± 8.74 | 69.64 ± 9.54 | 0.001 |
| Blood test |  |  |  |
| Creatinine (mg/dL) | 0.95 ± 0.11 | 0.94 ± 0.11 | 0.63 |
| Total cholesterol (mg/dL) | 168.41 ± 33.28 | 167.01 ± 31.15 | 0.42 |
| HDL-C (mg/dL) | 49.42 ± 9.57 | 48.86 ± 10.31 | 0.30 |
| LDL-C (mg/dL) | 103.98 ± 30.19 | 101.26 ± 27.10 | 0.07 |
| Triglycerides (mg/dL) | 97.02 ± 67.71 | 99.13 ± 81.07 | 0.60 |
| Fasting glucose (mg/dL) | 93.13 ± 9.01 | 92.36 ± 10.27 | 0.14 |
| Current tobacco smoking | 253 [43.8] | 356 [43.9] | 0.98 |
| Exercise performance |  |  |  |
| 3-KM running (seconds) | 864.88 ± 82.63 | 853.03 ± 86.09 | 0.01 |

Continuous variables are expressed as mean ± SD (standard deviation), and categorical variables as n [%]

Abbreviations: HDL-C, high-density lipoprotein cholesterol; KM, kilometer; LDL-C, low-density lipoprotein cholesterol.
